# Comparison of Adhesion and Proliferation of Human Gingival Fibroblasts on Acellular Dermal Matrix with and without Low Level Diode Laser Irradiation, an *in vitro* Study

**DOI:** 10.30476/DENTJODS.2021.87281.1251

**Published:** 2022-06

**Authors:** Orod Taghva, Shirin Amini Sedeh, Fatemeh Ejeian, Shahram Amini

**Affiliations:** 1 Postgraduate Student, Dept. of Periodontics, Faculty of Dentistry, Isfahan (Khorasgan) Branch, Islamic Azad University, Isfahan, Iran; 2 Dept. of Periodontics, Faculty of Dentistry, Isfahan (Khorasgan) Branch, Islamic Azad University, Isfahan, Iran; 3 Dept. of Animal Biotechnology, Cell Science Research Center, Royan Institute for Biotechnology, ACECR, Isfahan, Iran

**Keywords:** Acellular Dermal, Fibroblasts, Lasers, Cell Proliferation

## Abstract

**Statement of the Problem:**

In recent years, regeneration of periodontal soft tissues in the reconstruction of periodontal defects and the finding of suitable membranes and graft materials for the placement of autogenous grafts have been of great interest in various studies. In this regard, the proliferation and adhesion of regenerative cells are two linchpins of the complete regenerative process.

**Purpose:**

This study aimed to evaluate the effects of low-level laser beams on the attachment and the proliferation of human gingival fibroblasts in the presence of acellular dermal matrix (ADM).

**Materials and Method:**

All the experiments were conducted compared to tissue culture plate in four groups as follows: (1) Fibroblast+ADM+laser, (2) Fibroblast+ADM+ no laser, (3) Fibroblast + laser radiation, and (4) Fibroblast+ no laser. In this experimental study, the primary attachment was evaluated by passing 8h from seeding of 5×10^5^ gingival fibroblasts with or without a single dose (15.6 J/cm^2^) of laser radiation. Cell proliferation rate was also examined at 24, 48, and 72 hours after cell culture, following exposure to 5.2 J/cm^2^ of laser at each day of examination. Thereafter, fibroblasts were incubated under the normal culture condition (at 37°C, 5% CO_2_) in high glucose Dulbecco's Modified Eagle's medium (DMEM) medium supplemented with 10% fetal bovine serum, 1% glutamax, and 1% penicillin/streptomycin. Subsequently, the cellular viability was assessed on each time point using MTS calorimetric assay. The obtained data were statistically analyzed by applying ANOVA and Tukey tests.

**Results:**

There was a significant difference among the means of these four groups in terms of the proliferation of fibroblasts at 24, 48 and 72 hours (*p*< 0.001). Moreover, there was no significant difference among the means of two groups in terms of fibroblastic attachment in 8 hours (*p*< 0.2). The fibroblast group has shown the highest proliferation rate among all groups after laser radiation.

**Conclusion:**

It was indicated that the laser radiation increases the fibroblast cell proliferation. Accordingly, although this increase was higher in the fibroblast group alone compared to the fibroblasts cultured on acellular dermal matrix, the laser radiation did not significantly increase the attachment of fibroblast cells to acellular dermal matrix.

## Introduction

Autogenous gingival grafts are commonly used in periodontal surgeries. Since these grafts are prepared from the individual's mouth, they require an additional surgery that consequently causes tissue constraints in the donor area [ 1 ]. It is noteworthy that acellular dermal matrix (ADM) has been introduced as an alternative for autogenous grafts in periodontology [ 2 ]. Correspondingly, it has some advantages over sub epithelial connective tissue grafts, including the presence of adequate amounts of substance, the reduced surgical time and discomfort, removal of the donor area, and a reduction of postoperative complications [ 3 ]. Although the absence of cells and blood vessels lead to slower maturation of the tissue vessels as compared to the sub epithelial connective tissue graft and contrary to the autogenous graft, re-establishment of blood flow mostly depends on the receptor area [ 2 ]. Therefore, in order to solve the above-mentioned problems, the addition of fibroblasts to the ADM might be effective on achieving a faster regeneration, the improved tissue maturity, and the reduced tissue contraction [ 4 - 5 ]. Overall, fibroblasts play an important role during the wound healing process. Furthermore, by considering their presence and rapid proliferation in the wounded area, these cells could accelerate the regeneration process and the ADM acts as a scaffold for host cells, which consequently causes the migration, binding, and proliferation of fibroblasts on its surface [ 6 ]. Notably, collagen will be protected due to the controlled increase in its amount and by considering its normal placement in the gingival tissue after periodontal surgeries [ 7 ]. Gingival connective tissue fibroblasts by producing macromolecules like collagen play a very important role in periodontal treatments and implants. Therefore, considering this capacity of fibroblasts as well as their ability in producing elastin, fibronectin, proteoglycans, enzymes, and growth factors, their important roles in wound healing and gingival tissue protection, especially after periodontal surgeries or conservative periodontal treatments, are regarded [ 8 - 9 ]. Nowadays, the Low-level laser therapy is used in periodontal surgeries for some purposes, including controlling inflammation of the inflamed tissue, accelerating the wound healing, reducing pain, and controlling chronic mucosal diseases [ 10 - 11 ]. These lasers have also shown to encompass bio stimulation effects on cell proliferation in the medium [ 12 ]. Generally, a low-power diode laser can deeply penetrate into the periodontal tissues and finally produce some beneficial effects [ 13 ]. In addition, it can also control inflammation of the inflamed periodontal tissues as well as the regeneration of them following performing surgical and non-surgical treatments [ 14 , 10 ]. In this regard, in a study conducted on the effects of the low-power diode laser radiation on the human gingival fibroblast proliferation rate, Kreisler *et al*. [ 15 ] have shown that the cells exposed to radiation had a significantly higher proliferation activity compared to the control group. Moreover, in a study by Ren *et al*., [ 16 ] it was shown that the low-power diode laser had a positive effect on the increased proliferation of fibroblasts, osteogenic differentiation, and regulation and adjustment of cellular inflammation by changing the gene expression as well as the release of growth factors, bone remodeling markers, and inflammatory mediators. Since the use of different types of tissue grafts and lasers is common in periodontal treatments, it can be concluded that the greater the adhesion and proliferation of fibroblasts on the ADM are, the more likely soft tissue regeneration would be. Regarding the effectiveness of low-power lasers on the tissue regeneration and the limited studies performed on the adhesion and proliferation of fibroblasts on the ADM in case of using a low-power diode laser, the present study aimed to investigate the effect of the low-power diode laser on the fibroblasts’ proliferation and adhesion on the ADM under in vitro condition.

## Materials and Method

### Cell culture and expansion

In this experimental study, human gingival fibroblasts, which were previously characterized, were obtained from Royan institute for biotechnology [ 17 ]. The cells at passage 3 were expanded under the normal culture condition (at 37°C and 5% CO_2_) in high glucose Dulbecco's Modified Eagle's medium (DMEM) supplemented with 10% fetal bovine serum (FBS), 1% glutamax, and 1% penicillin/streptomycin (pen/str). Notably, all the components were purchased from Gibco (Ireland). The medium was also replaced three times per week and the cells were passaged after reaching 80-90% confluence. For further analyses, gingival fibroblasts were used at passage 5.

### Preparation of ADMs

Before cell culturing, ADMs (SureDerm 0.6-0.99mm, Korea) were prepared in terms of the manufacture’s instruction and cut into 49mm^2^ pieces precisely. Afterward, the ADM slices were placed in 96-well plates to create the optical isolation during laser irradiation process. 

### Cell seeding on ADMs

Next, the cells were harvested and 5 × 10^5^ of cells were seeded on each ADM pieces for examining the cell adhesion and proliferation with/without laser irradiation. Moreover, the cells were cultured on tissue culture plates (TCPs) under the same condition as the positive control. Notably, the four experimental groups were labeled as follows (n=4): (1) Fibroblast + ADM + laser radiation, (2) Fibroblast + ADM + no laser radiation, (3) Fibroblast + laser radiation and (4) Fibroblast+ no laser radiation. 

### Laser irradiation and Cell attachment analysis

In order to investigate the effect of laser radiation on primary attachment of fibroblasts to ADMs, the cultured cells in this study were exposed to a single dose continuous wave (CW) laser radiation (Doctor Smile Diode 808 nm. Italy) for 30 seconds (Bean spot 0.38cm^2^) 0.2 MW laser (totally 15.6 J/cm^2^). Following the incubation for 8h under the normal culture condition (at 37_°C_, 5% CO_2_) MTS assay (Cell Titer 96® Aqueous One Solution, Promega, Leiden, Netherlands) was applied in terms of the manufacturer’s instruction. Briefly, the MTS solution was added to the fresh culture media (1:10) and then incubated for 3.5 hours under the culture condition. Afterward, the optical absorbance of formazan dye, produced by mitochondrial dehydrogenase enzymes in living cells, was evaluated at the wavelength of 490 nm using the ELISA reader.

### Laser irradiation and Cell proliferation analysis

To evaluate the proliferation rate of fibroblasts on the ADM, the cells were assessed in three time intervals during 72 hours. The CW laser with the dose of 5.2J/ CM^2^ was irradiated (Beam spot 0.38cm^2^) for three consecutive days (a total dose of 15.6 J/ cm^2^) with 24-h interval. In order to monitor the proliferation rate of the cells during the treatment time, viability of the cultured cells was assessed using the MTS assay by passing 24, 48 and 72 hours from seeding and after receiving the radiation dose.

Data analysis was then performed using ANOVA and Tukey tests in SPSS 22. In addition, *p*< 0.05 was considered as the statistical significance level.

## Results

There was a significant difference among the means of the four groups in terms of fibroblast proliferation at 24, 48 and 72 hours (*p*< 0.001) (Table 1). Pairwise-comparison of these groups has also shown a significant difference among all these groups in terms of the rate of fibroblast cell proliferation at the 24-hour period (*p*< 0.01). Moreover, there was no significant difference between the fibroblast+no laser radiation and fibroblasts+no laser+ADM groups in the same period (*p*= 0.77) (Table 2). Additionally, we have found no significant difference among these groups in terms of fibroblast proliferation rate at 48-hour period (*p*< 0.01). besides, there was no statistically significant difference between the fibroblast + no laser radiation and fibroblasts+ no laser radiation + ADM groups in terms of fibroblast proliferation rate (*p*= ) (Table 3). There was also a statistically significant difference among these groups in terms of fibroblast proliferation rate at 72-h period (*p*< 0.001) (Table 4).

**Table 1:** The Mean±SD of proliferation of fibroblasts after 24, 48, 72 .ADM: acellular dermal matrix

	Fibroblast+no laser	Fibroblast+laser	Fibroblast+ADM+no laser	Fibroblast+ADM+laser	*p* Value
	Mean ± sd	Mean ± sd	Mean ± sd	Mean ± sd	
24 hour	0.215±0.04	0.31±0.037	0.222±0.043	0.038±0.017	0.001
48 hour	0.176±0.012	0.62±0.035	0.209±0.048	0.021±0.011	0.001
72 hour	0.133±0.016	0.729±0.033	0.198±0.009	0.073±0.016	0.001

**Table 2:** The pairwise comparison of the groups study groups fibroblast proliferation in the 24-hour, ADM: acellular dermal matrix

	Fibroblast+no laser	Fibroblast+laser	Fibroblast+ADM+no laser	Fibroblast+ADM+laser
Fibroblast+ no laser	-	0.001	0.77	0.001
Fibroblast + laser	0.001	-	0.001	0.001
Fibroblast+ADM+no laser	0.77	0.001	-	0.001
Fibroblast+ADM+laser	0.001	0.001	0.001	-

**Table 3:** The pairwise comparison of the groups study groups fibroblast proliferation in the 48-hour, ADM: acellular dermal matrix

	Fibroblast+no laser	Fibroblast+laser	Fibroblast+ADM+no laser	Fibroblast+ADM+laser
Fibroblast+ no laser	-	0.001	0.14	0.001
Fibroblast + laser	0.001	-	0.001	0.001
Fibroblast+ADM+no laser	0.14	0.001	-	0.001
Fibroblast + ADM + laser	0.001	0.001	0.001	-

**Table 4:** The pairwise comparison of the groups study groups fibroblast proliferation in the 72-hour, ADM: acellular dermal matrix

	Fibroblast+no laser	Fibroblast+laser	Fibroblast+ADM+no laser	Fibroblast+ADM+laser
Fibroblast+ no laser	-	0.001	0.001	0.001
Fibroblast+laser	0.001	-	0.001	0.001
Fibroblast+ADM+no laser	0.001	0.001	-	0.001
Fibroblast+ADM+laser	0.001	0.001	0.001	-

**Figure 1:**
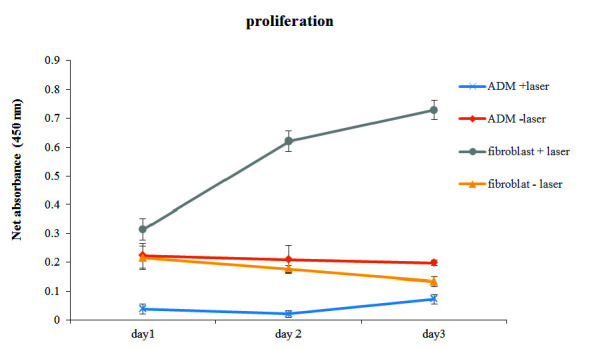
The changes of proliferation at 24, 48 and 72 hours, ADM: acellular dermal matrix

Based on the one-way analysis of variance test within the fibroblast group, there was a significant difference between fibroblast+ no laser radiation over time from 24 to 72 hours (*p*= 0.001). Furthermore, in the fibroblast group, there was a significant difference between fibroblast+ laser radiation over time from 24 to 72 hours (*p*= 0.001), as well as a significant difference between Fibroblast+ADM+laser over time from 24 to 72 hours (*p*= 0.001). However, in the fibroblast group, there was no statistically significant difference in the fibroblasts+ no laser radiation + ADM group between the two-time intervals of 24 and 72 hours (*p*= 610) (Table 5 Figure 1). There was no significant difference among the four groups in terms of the fibroblast adhesion after 8 hours (*p*= 0.2) (Table 6).

**Table 5:** Comparison the mean of fibroblastic proliferation in experimental groups over 24 to 72 hours, ADM: acellular dermal matrix

	24 hour	48 hour	72 hour	*p* Value
	Mean ± sd	Mean ± sd	Mean ± sd	
Fibroblast+no laser	0.215±0.04	0.176±0.012	0.133±0.016	0.001
Fibroblast + laser	0.31±0.037	0.62±0.035	0.729±0.033	0.001
Fibroblast+ADM+no laser	0.222±0.043	0.209±0.048	0.198±0.009	0.61
Fibroblast+ADM+ laser	0.038±0.017	0.021±0.011	0.073±0.016	0.001

**Table 6:** The mean of fibroblast adhesion after 8 hours, ADM: acellular dermal matrix

	Fibroblast+no laser	Fibroblast+laser	Fibroblast+ADM+no laser	Fibroblast+ADM+laser	*p* Value
	Mean ± sd	Mean ± sd	Mean ± sd	Mean ± sd	
8 hour	0.16 ± 0.021	0.139 ±0.024	0.172 ±0.01	0.141 ± 0.026	0.2

## Discussion

The results of the investigation of the fibroblast proliferation rate at the first, second, and third days showed that the fibroblast+ laser radiation group had a higher proliferation rate compared to the fibroblast+ no laser radiation group, which is consistent with the results of other studies [ 18 - 23 ]. However, in previous studies, different low-power lasers have been used, and regardless of the type of laser, there has been an increase in the population of gingival fibroblasts in most of them [ 15 , 20 ].

The results of other studies performed on the fibroblast absorption spectrum showed that the absorption occurs, especially at 800 to 830 nm wavelengths [ 15 , 24 - 25 ]. Generally, the molecular absorption of the radiated laser is a prerequisite for any biological cellular effect, which is mostly dependent on the laser dose and wavelength [ 26 ]. For example, 830-nm GaAlAs laser radiation can have some therapeutic effects on gingivitis and periodontitis associated with bacterial infections by the inhibition of the production as well as the expression of the prostaglandin E2 (PGE2) and interleukin-1 beta gene [ 26 ]. Accordingly, this wavelength can also inhibit the plasminogen activator [ 28 ]. The results of the present study show that 808-nm diode laser radiation has a significant stimulatory effect on human gingival fibroblast proliferation rate, which is consistent with the results of the Kreisler *et al*.'s study [ 19 ]. Notably, the laser investigated in the present study was radiated at the dose of 5.2.J/cm^2^/a day (a total of 15.2J/cm^2^) for three consecutive days. The results of some studies have also indicated that low-level laser therapy at different energy densities (up to 4J/cm^2^) has proliferation stimulatory effects on the gingival fibroblast proliferation. In addition, it was shown that those lasers with energy densities above the mentioned level would induce some inhibitory properties, which were not observed in the present study [ 29 - 30 ]. 

In another study, the proliferation stimulatory effects of the laser were reported at the energy densities of 2-8J/cm^2^ [ 15 ]. Finally, Ren *et al*. [ 16 ] in their review study reported such stimulatory effects on the gingival fibroblast proliferation in case of using a laser with the energy density of 0.5-16J/cm^2^. The results of the investigation of the fibroblast proliferation showed that the laser fibroblasts+ laser radiation group had a higher proliferation rate compared to the fibroblasts+ no laser radiation group every three days, which is consistent with the results of the Kreisler *et al*. [ 15 ] study. However, these effects are not persistent and the cell proliferation rate has decreased after the completion of laser radiation. Correspondingly, this can be attributed to the gradual reduction of the laser effect or the fibroblasts reaching the proliferation saturation point after 3 days. However, Frozanfar *et al*. [ 23 ] showed that cell proliferation started after the first day, which significantly increased at the second and third days. The increased fibroblast proliferation caused by the laser radiation can also be associated to the production of autocrine growth factors. In another study, Yu *et al*. [ 31 ] reported the increased concentration of basal fibroblast growth factor (bFGF), which is consistent with the results of the Asl *et al*.'s study [ 32 ], which showed that basic fibroblast growth factor (BFGF), is as an important factor for periodontal tissue regeneration. Moreover, Kreisler *et al*. [ 15 ] reported some similar results using 809-nm diode laser. There was no significant difference between fibroblast+ no laser irradiation and fibroblast+ no laser+ ADM groups in terms of the proliferation rate at the first and second days. However, this difference was significant at the third day in such a way that fibroblast+ no laser+ ADM group exhibited more proliferation rate that is consistent with the results of some other studies [ 4 - 5 ]. In the present study, the fibroblast + laser radiation group had a higher proliferation rate compared to the other three groups, including the fibroblast+ ADM+ laser radiation group in all three-time intervals. Nevertheless, the fibroblast+ no laser radiation+ ADM group exhibited a higher proliferation rate as compared to the fibroblast+ laser+ ADM group, but only at the third day. In this regard, it was observed that the laser radiation might cause some changes in the structure of ADM or inhibit the fibroblast proliferation by creating specific conditions. It is noteworthy that there was no significant difference among the studied groups regarding the fibroblast adhesion; however, there has been no study conducted on the effect of low-power diode laser on the fibroblastic adhesion on the ADM yet. Kreisler *et al*. [ 33 ] investigated the cellular effects of diode laser on the adhesion of periodontal ligament cells on dental surfaces. Finally, they reported that the use of diode lasers would have no significant positive effect on the adhesion of periodontal ligament cells [ 33 ], which is consistent with the results of the present study due to the differences in the design of these two studies. In another study performed on the adhesion and survival of gingival fibroblasts on ADM during 7, 14 and 21-day time periods, Rodrigues *et al*. [ 34 ] have shown that fibroblast seeding on ADM for 14 days could create appropriate conditions for the adhesion of fibroblasts and their release on the matrix; however, their migration into the matrix is limited.

## Conclusion

Laser radiations have several significant effects on in creasing the human gingival fibroblast proliferation rate, which promises a new method in the field of regeneration of periodontal tissues. Although the above-mentioned cells showed the increased proliferation rate under the laser radiation, this increase was lower in the ADM group compared to the fibroblast group alone. On the other hand, in the present study, the laser radiations had a little effect on the adhesion of gingival fibroblast cells, either alone or near ADM.

## Conflict of Interest

The authors report no conflicts of interest.
